# Report of a Leprosy case in Singapore: an age-old disease not to be forgotten in developed countries with low-prevalence settings

**DOI:** 10.1099/acmi.0.000014

**Published:** 2019-04-05

**Authors:** Yen Ee Tan, Yi Wei Yeo, David Jia Qiang Ang, Michelle Mei Fung Chan, Shiu Ming Pang, Li-Hwei Sng

**Affiliations:** 1 Department of Microbiology, Singapore General Hospital, 20 College Road 169856, Singapore; 2 Department of Dermatology, Singapore General Hospital, 20 College Road 169856, Singapore; 3 MOH Holdings Pte Ltd, 1 Maritime Square #11-25 HarbourFront Centre 099253, Singapore; 4 Department of Anatomical Pathology, Singapore General Hospital, 20 College Road 169856, Singapore

**Keywords:** leprosy, Singapore, developed countries, low prevalence, diagnostics, healthcare worker

## Abstract

**Introduction:**

Leprosy is rarely reported in developed countries with low-prevalence settings. Its diagnosis may be missed due to its low frequency in non-endemic regions, as well as its long incubation period. The report describes an imported leprosy case of a healthcare worker in Singapore.

**Case presentation:**

A Filipino nursing personnel presented with a persistent non-tender erythematous plaque over his right upper back for many years despite topical treatment. He had the lesion before coming to Singapore but decided to seek medical consultation only after the lesion progressed with new erythematous papules developing over his face, trunk and upper limbs. Punch biopsies of skin lesions revealed fite-positive bacilli, which were identified to be *Mycobacterium leprae* by GenoType LepraeDR v1 assay (Hain LifeScience, Germany). No mutation was detected at *rpoB* (rifampicin), *gyrA* (ofloxacin) and *folP1* (dapsone) gene targets. He was started on multi-drug therapy and responded to the treatment. The only prolonged close contact he had was his housemate who was screened and given a single dose of rifampicin as chemoprophylaxis.

**Conclusion:**

In non-endemic settings, awareness is crucial in diagnosing leprosy. The availability of molecular testing and multi-disciplinary management are essential in the confirmation and control of this disease of public health importance.

## Introduction

Leprosy, also known as Hansen’s disease, has been classified as a neglected tropical disease (NTD) by the World Health Organization (WHO) [1]. It is an infectious disease caused by *Mycobacterium leprae* and *Mycobacterium lepromatosis* and is often seen in the remote rural areas with tropical or subtropical conditions. Leprosy is rarely reported in developed countries [2], especially in low-prevalence settings such as Singapore. With increasing mobility of skilled migrants across the globe and international travel, however, patients with leprosy may present anywhere. Singapore is the melting pot of Asia with an influx of foreigners from nearby countries, which are endemic for leprosy. Though rare here, medical practitioners need to be aware of the possibility of leprosy and its presentation to avoid misdiagnosis. A high degree of clinical suspicion and the availability of laboratory diagnostic are required to make a correct diagnosis in this age-old disease of public health importance. Here, we report an imported case of leprosy in a healthcare worker in Singapore, which was confirmed by laboratory diagnosis. Informed consent for publication was obtained from the patient.

## Case Report

A Filipino male in his 30s presented with a persistent non-tender erythematous plaque over his right upper back for many years. It began as a faint erythema that slowly developed into a ring-like plaque lesion. It was non-pruritic in nature and the plaque did not respond to topical antifungal treatment. He had this skin lesion prior to arriving in Singapore but decided to seek medical attention as the lesion progressed with new erythematous papules developing over the face, trunk and upper limbs in the past 2 months. There was no prior trauma or irritants applied to the skin and the patient did not complain of any systemic symptoms of fever, loss of weight and night sweat. There was no numbness or weakness and he did not have another co-existing medical condition. He denied ingestion of medication and sexual contact with a commercial sexual worker. The patient came to Singapore 7 years ago to work as nursing personnel and travelled back to the Philippines once to twice a year to visit his family. His housemate and family members did not have any skin complaint.

**Fig. 1.**
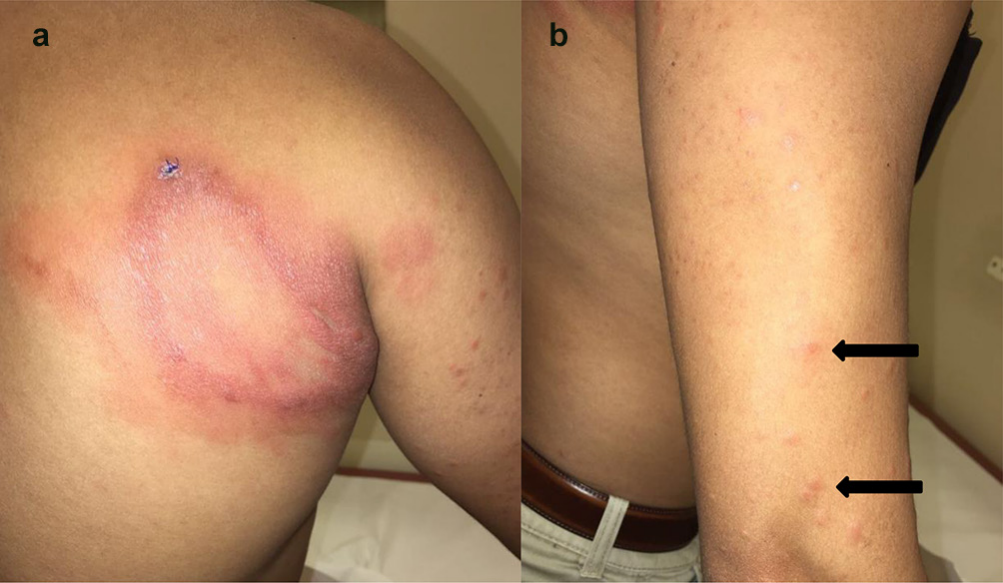
(a) A large well demarcated annular plaque of 10 by 7.5 cm over the right upper back, which extended to the axilla. (b) Multiple monomorphic papules of 0.5 to 1 cm in size over the right upper limb (bold arrows).

**Fig. 2.**
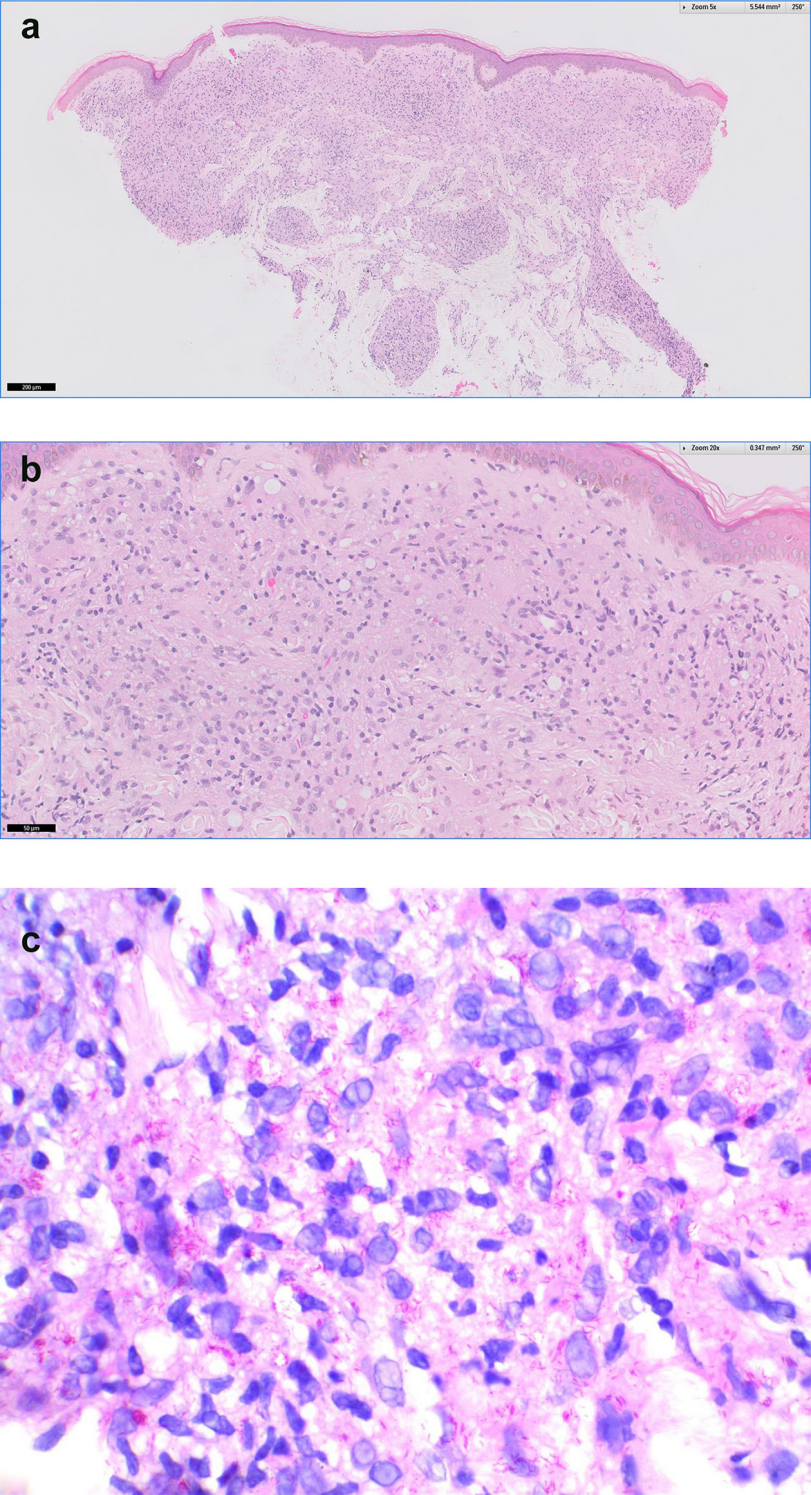
(a) Skin punch biopsy from the right upper back plaque (H&E). (b) There is a diffuse superficial dermal infiltrate of epithelioid histiocytes without well-formed granulomas. Foamy cytoplasm was seen in only a portion of the histiocytes (H&E). (c) Fite-positive bacilli were observed within the inflammatory infiltrate (Fite stain).

On physical examination, there was a large well demarcated annular plaque of 10 by 7.5 cm over the right upper back, which extended to the axilla (Fig. 1a). It was erythematous, infiltrated and not scaly. There were also multiple monomorphic papules of 0.5 to 1 cm in size over the upper limbs (Fig. 1b), face, proximal aspect of lower limbs and the gluteal region. Punch biopsies of the skin lesions were taken from the plaque at the back and one of the papules at the upper limb. Microscopic examination of the skin biopsies revealed a diffuse superficial dermal infiltrate of epithelioid histiocytes without well-formed granulomas surrounded by lymphocytes or any multinucleate Langhans giant cells. Foamy cytoplasm was seen in only a portion of the histiocytes and was subtle (Fig. 2a, b). Fite-positive bacilli were identified within the inflammatory infiltrate (Fig. 2c). All the controls demonstrated appropriate reactivity. In view of the histopathological findings, the microbiologists were consulted and deparaffinized samples from the skin lesions were sent for non-tuberculous mycobacteria (NTM) and leprosy molecular testing, which were performed using commercially available line-probe assays INNO-LiPA Mycobacteria v2 (Innogenetics, Belgium) and GenoType LepraeDR v1 (Hain LifeScience, Germany), respectively. The result was positive for *M. leprae* with no mutation detected at *rpoB* (rifampicin), *gyrA* (ofloxacin) and *folP1* (dapsone) gene targets by the assay.

The patient was followed up with the history and physical examination revisited. He came from a rural area with rice paddy fields and subsequently moved to the city to work. In Singapore, he worked in the operating theatre and assisted the anesthetist in intubation. A surgical mask and gloves were worn in the workplace at all times. He did not recall coming into contact with people suffering from leprosy. A detailed physical examination also revealed diminished sensation at the centre of the large annular indurated plaque over the right upper back and the ulnar nerves were mildly thickened bilaterally. Coarse facies was not noted. The patient was diagnosed to have borderline lepromatous leprosy and was counselled for the disease with the management explained. He was subsequently treated with multi-drug therapy (MDT) consisting of rifampicin, clofazimine and dapsone and responded to the treatment. The plaque and papules flattened with minimal induration. The case was notified to the relevant health authority and the infection control department in the workplace. Screening of his housemate was advised and he was offered a single dose of rifampicin for chemoprophylaxis.

## Discussion

Leprosy is not a disease of the past. It has not been eradicated completely and still poses public health concern in modern-day societies. In 2016, WHO launched its ‘Global Leprosy Strategy 2016–2020: Accelerating towards a leprosy-free world’ campaign to reinforce and strengthen the efforts for leprosy control [3]. Though less common, it is still important to maintain vigilance and diagnostic capabilities for leprosy in the developed countries with lower incidence as the world becomes more connected with people travelling widely and migrants moving from high-risk rural areas to the more urbanized cities. In Singapore, leprosy is notifiable and the incidence of this disease remains low with seven reported cases in 2016 [4]. Worldwide, WHO reported a total of 214 783 new cases of leprosy, which involved 145 countries or territories; 9 of which were from the Southeast Asian region and 30 from the Western Pacific region. Notably, the Philippines and India reported 1721 and 135 485 cases in 2016, respectively. The highest burden in the world is located at India, which consisted of more than half of the world’s leprosy cases [1]. Drug-resistant cases have also been reported in a surveillance performed by WHO [5]. In the United States (US), leprosy is emerging with autochthonous cases reported [6]. This is worrisome and the awareness and effort to control leprosy must be strengthened in the developed countries to prevent pockets of endemicity from emerging. It would be even harder to eradicate the disease once it gained a foothold in the non-endemic region.

Although the incidence of leprosy is low in Singapore, clinical vigilance and education remains important to avoid misdiagnosis. Heightened awareness is necessary as Singapore is surrounded by countries that are endemic for leprosy. The disease can present anywhere these days with the increase in international travel and migration of skilled workers. This case highlights the importance of clinical suspicion especially in a patient whose native country is endemic for leprosy. It is important to note that *M. leprae* has a long incubation period of up to 2 decades [3]. Hence leprosy is still a possible differential in patients who have migrated for a number of years and presents with atypical rashes that do not respond with topical treatment as seen in this case. The case also illustrated the importance of a multi-disciplinary team effort, which involved dermatologists, histopathologists, microbiologists and infection control practitioners in the diagnosis and management of leprosy.

Laboratory diagnosis of leprosy can be challenging in a low-prevalence setting as most laboratories do not have the capability to test for the pathogen. Maintaining laboratory expertise can be costly. Although the diagnosis of leprosy is largely based on clinical findings and histology, the availability of molecular testing as a diagnostic tool can be useful in confirming the clinical suspicion. Slit-skin smear, a semi-quatitative test, may serve as an ancillary tool in the diagnosis, classification and monitoring of treatment response in leprosy cases; but skilled technicians are required to perform this technique for reliable results [7].

Fite stain, on the other hand, is a useful special stain, which is typically used in the histopathologic examination of fixed tissue to examine for acid-fast organisms such as *M. leprae *. As leprosy bacilli is less acid and alcohol fast than tubercle bacilli, 5% sulphuric acid is used in the decolourization process in place of an acid–alcohol solution. The process of deparaffinization is also a delicate procedure. A mixture of peanut oil and xylene is used to protect the cell wall from solvent and the acid-fastness of *M. leprae* [8].

*M. leprae* is not cultivable *in vitro* except in animal models. It is also not viable to perform susceptibility testing in a routine diagnostic laboratory setting as the conventional method is to inoculate the bacteria into a mouse footpad that requires a 1 year duration due to the long replication time of the pathogen [7, 9]. It may only be performed in specialized laboratories with animal facilities and is useful in viability studies, vaccine development and the detection of new mutations [7, 9]. Molecular techniques improve the detection rate of leprosy in the laboratory setting greatly. Commercially available assays such as GenoType LepraeDR (Hain LifeScience v1, Germany) are useful in providing rapid identification and resistance profile genotypically [9]. It is a line-probe assay that utilizes DNA probes in the detection of *M. leprae* and resistance to rifampicin, ofloxacin and dapsone in the same setting. This allows prompt diagnosis to halt transmission and appropriate treatment to be initiated, which decreases the risk of long-term sequelae. However, the test is not cheap (~SGD 200) and can only be performed from acid-fast bacilli (AFB) positive skin biopsies. Certain sub-types and resistance not covered by the probes may potentially be missed [10]. The advent of newer technology such as next generation sequencing (NGS) will have an evolutionary role in the diagnosis and control of leprosy. The knowledge of whole genome sequences will enable molecular drug susceptibility to be performed and potentially more resistance gene targets to be identified. It will also allow detailed phylogeny to be performed, which will be useful in the control of leprosy at large. Stefani *et al.* have successfully utilized NGS to distinguish reinfection from relapse in patients with recurrent leprosy and demonstrated that treated leprosy cases can be reinfected by another strain in endemic areas [11]. Such an application reveals how powerful NGS can be in evaluating transmission dynamics and outcome.

Leprosy control is an ongoing effort involving education, contact tracing and chemoprophylaxis. Droplet transmission is the likeliest mode of spread of the disease. WHO recommended single-dose rifampicin (SDR) as the chemoprohylactic intervention for contacts of leprosy cases [12]. The Public Health England (PHE) guideline mentioned that household contacts should be traced and SDR may be considered if the index case has multi-bacillary leprosy. Even so, only about 5% of spouses of lepromatous cases develop leprosy, which indicated that infection control precautions are rarely required. Untreated leprosy patients do not require isolation if they are admitted to healthcare facilities. Only standard precautions are needed if they have open wounds or undergoing surgical procedures such as skin biopsies. *M. leprae* in the tissues are generally scanty and most are intracellular, degenerate and non-viable [13]. It was fortunate that our patient wore a surgical mask and gloves in his workplace, which minimized the risk of transmission.

To conclude, leprosy is still present in Singapore and it is important for clinicians to remain vigilant in order to diagnose the disease. Leprosy can happen anywhere including developed countries due to the high mobility of populations in current times and its long incubation period. Maintaining laboratory expertise remains relevant and molecular diagnostics expedite the testing in this slow-growing organism. A multi-disciplinary team effort is required to control this disease of public health importance.
